# Knowledge, attitudes, and practices of polio vaccination among mothers attending vaccination sites in Gaza during the conflict

**DOI:** 10.3389/fped.2025.1569650

**Published:** 2025-05-30

**Authors:** Abdel Hamid El Bilbeisi, Amany El Afifi, Samer Abuzerr, Isra Kanan Alwahedy, Saja Kanan Alwahedy

**Affiliations:** ^1^ Department of Clinical Nutrition, Faculty of Applied Medical Sciences, Al-Azhar University of Gaza, Gaza, Palestine; ^2^ Department of Nutrition, School of Medicine and Health Sciences, University of Palestine, Gaza, Palestine; ^3^ Faculty of Pharmacy, Al-Azhar University of Gaza, Gaza, Palestine; ^4^ Department of Medical Sciences, University College of Science & Technology, Khan Younis, Gaza, Palestine; ^5^ Faculty of Medicine, Al-Azhar University of Gaza, Gaza, Palestine

**Keywords:** attitudes, Gaza war, knowledge, mothers, polio vaccination, practices

## Abstract

**Objective:**

This study aims to evaluate the knowledge, attitudes, and practices (KAP) related to polio vaccination among mothers attending vaccination sites in Gaza during the ongoing conflict.

**Methods:**

A cross-sectional survey was conducted from 1 to 12 September, 2024, targeting a representative sample of 474 mothers from ten primary healthcare centers across the governorates of Gaza. Participants were randomly selected using a cluster sampling method. Data on socio-demographics and KAP towards polio vaccination were collected through a validated, interview-based questionnaire. Statistical analysis was performed using SPSS version 26.

**Results:**

Of the 474 mothers surveyed, 44.7% were between 26 and 30 years old. Around 35.4% had a primary-level education, 48.1% were employed, 48.5% had two to three children, and 49.4% had children aged between one day and less than twelve months. The majority of mothers exhibited good knowledge (85.7%), positive attitudes (86.1%), and good practices (67.1%) regarding childhood vaccination. Knowledge scores were significantly associated with the mother's age, number of children, and the children's age. Attitude scores were significantly related to the mother's age and number of children (*P* < 0.05 for all). However, no significant relationship was observed between socio-demographic factors and practice scores.

**Conclusion:**

Most mothers demonstrated good knowledge and positive attitudes towards polio vaccination, although only about two-thirds reported good vaccination practices. Younger mothers and those with younger children had better knowledge, while middle-aged mothers expressed more positive attitudes. Importantly, socio-demographic factors were not linked to practice levels. The relatively low adherence to vaccination practices requires further attention.

## Introduction

Vaccination remains one of the most effective tools in combating infectious diseases, saving millions of lives globally each year. Immunization prevents severe illness, reduces mortality rates, and contributes significantly to public health. The World Health Organization (WHO) estimates that vaccinations prevent around 2–3 million deaths annually, illustrating their profound impact on global health systems (1). However, despite the widespread availability of vaccines and comprehensive immunization programs, a significant number of children around the world still do not receive their essential vaccinations, particularly in low- and middle-income countries (LMICs) (2). The reasons for this range from logistical challenges, socio-economic barriers, and a lack of public confidence in vaccines to misinformation and cultural resistance to immunization programs.

Parental perspectives play a crucial role in childhood vaccination. Parents are the primary decision-makers when it comes to their children's health, and their knowledge, attitudes, and beliefs about vaccines significantly influence their children's vaccination status. While some parents fully support immunization programs, others express doubts or fears due to misinformation, medical concerns, or philosophical objections (3). These attitudes are often shaped by a range of factors, including religious beliefs, personal experiences, or mistrust in the healthcare system. Misunderstandings about the safety and necessity of vaccines can lead to vaccine hesitancy, which has become a growing issue not only in developed countries but also in underdeveloped regions where healthcare systems are already strained (4, 5). In some cases, parents who are not confident in the benefits of vaccination delay or avoid vaccinating their children altogether, putting entire communities at risk, particularly during outbreaks of vaccine-preventable diseases (3). Good practices refer to behaviors and actions taken by mothers to ensure their children receive timely and complete vaccinations. This includes attending scheduled immunization appointments, following up with booster doses, keeping vaccination cards updated, and actively seeking information or services related to child immunization (4). These practices are essential for achieving high coverage rates and protecting communities from vaccine-preventable diseases, especially in vulnerable settings like Gaza.

To ensure the continued success of immunization programs, it is essential to regularly assess the knowledge, attitudes, and practices (KAP) of parents regarding vaccination. This allows healthcare systems to identify gaps in awareness and address misconceptions through targeted educational campaigns. In turn, these efforts can foster positive attitudes towards vaccination and increase compliance rates (6). Studies have shown that public health interventions, including awareness programs and educational initiatives aimed at improving vaccination knowledge, can significantly reduce vaccine hesitancy and increase immunization coverage (7). Periodic and ongoing assessment of public knowledge is crucial in ensuring that immunization programs remain effective, especially in countries where vaccine hesitancy or logistical challenges pose barriers to full coverage (8).

Immunization is particularly critical in conflict-affected areas, where healthcare infrastructure is often weakened, and disease outbreaks pose a greater threat to vulnerable populations. In such settings, public health systems are frequently overburdened, and access to healthcare services can be severely limited. The situation in the Gaza Strip, for example, presents unique challenges to immunization efforts. Conflict and political instability have led to disruptions in healthcare services, making it difficult for families to access vaccination programs (9–11). Furthermore, the psychological stress of living in a war zone may reduce parental compliance with vaccination schedules, either because of logistical difficulties or fears of visiting healthcare centers during periods of conflict (12).

Parents’ decision-making in such contexts becomes even more critical. Mothers, in particular, are often the primary caregivers responsible for ensuring that their children receive appropriate medical care (13). Therefore, understanding their KAP towards vaccinations is essential for improving immunization coverage in these areas. Targeted interventions aimed at increasing mothers’ awareness and addressing their concerns about vaccinations are likely to have a significant impact on vaccination rates, thereby reducing the spread of preventable diseases like polio.

Given the ongoing conflict and the precarious healthcare situation in Gaza, the current study seeks to assess the KAP of mothers regarding polio vaccination in Gaza's primary healthcare centers during the war. By identifying knowledge gaps, misconceptions, and attitudes that may hinder vaccination efforts, this research aims to provide insights that could inform future interventions designed to increase vaccination uptake in conflict-affected regions.

## Materials and methods

### Study design, period, and setting

This cross-sectional study was conducted from 1 to 12 September, 2024, amidst ongoing conflict, at ten primary healthcare centers across the Gaza Strip. These centers were selected from different governorates, ensuring representation from two centers per governorate.

### Study participants, sample size calculation, and sampling technique

The study focused on mothers who were attending immunization services at the selected vaccination sites during the study period. Eligible participants included mothers aged 18 and above who were attending follow-up visits for their children's polio vaccinations. Mothers with cognitive impairments or those critically ill during data collection were excluded from participation.

The total number of mothers frequenting the healthcare centers for immunization services was estimated to be 10,635, as reported by the Palestinian Central Bureau of Statistics (PCBS) in 2023 (14). In the present study, the representative sample size was calculated using the following formula: $n=\frac{N\cdot Z^{2}\cdot P\cdot (1-P)}{E^{2}\cdot (N-1)+Z^{2}\cdot P\cdot (1-P)}$ (15). Where: *N* = Total population size (10,635); Z = Z-value for 95% confidence (1.96); *P* = Estimated proportion (0.5); and E = Margin of error (0.05).

$$n=\frac{10,635\cdot {\left(1.96\right)}^{2}\cdot 0.5\cdot (1-0.5)}{{\left(0.05\right)}^{2}\cdot (10,635-1)+{\left(1.96\right)}^{2}\cdot 0.5\cdot (1-0.5)}$$

Accordingly, a representative sample of 474 mothers was randomly selected using a cluster sampling method (with a response rate 100%), which was adequate to detect statistically meaningful differences. A cluster sampling technique was employed to ensure practical and representative data collection given the constraints of conflict and accessibility. Clusters were defined based on the ten selected primary healthcare centers, with two centers chosen per governorate, representing the five governorates of the Gaza Strip (*N*orth Gaza, Gaza, Deir al-Balah, Khan Younis, and Rafah). Each center acted as a distinct cluster. Within each cluster, mothers were randomly selected from those attending polio immunization services during the data collection period. The sampling strategy was stratified by district (governorate) to ensure geographic representativeness across the Gaza Strip.

### Data collection tools

Data related to socio-demographic characteristics and the mothers’ knowledge, attitudes, and practices (KAP) regarding polio vaccination were collected using a validated, interview-based questionnaire. The questionnaire was designed to ensure comprehensive coverage of relevant topics, allowing for a thorough assessment of the participants’ perspectives.

### Assessment of knowledge, attitudes, and practices (KAP) towards polio vaccination

The KAP data was gathered using a structured questionnaire, previously validated by Almutairi et al., which comprised three main sections (16):

1. **Knowledge:** This section consisted of 10 items assessing the mothers’ understanding of polio vaccination. Responses were scored as “yes” (1 point) or “no” (0 points). Total knowledge scores ranged from 0 to 10, with scores categorized as “Poor” (0–4) and “Good” (5–10).
2. **Attitudes:** The attitude section contained 4 items, each scored as “negative” (0 points) or “positive” (1 point). Total attitude scores were categorized as “Negative” (0–1) or “Positive” (2–4).
3. **Practices:** The practices section also contained 4 items, with scoring similar to the attitude section. Practice scores were categorized as “Negative” (0–1) or “Positive” (2–4).

### Pilot study

A pilot study was conducted with a sample of 20 participants to test the questionnaire and data collection procedures. Based on the feedback from this pilot, necessary adjustments were made to the questionnaire to ensure clarity and accuracy in the main study.

### Statistical analysis

Data were analyzed using SPSS software (version 26). Descriptive statistics, including frequency and percentage distributions, were employed to summarize socio-demographic characteristics and KAP scores. To assess associations between categorical variables, the Chi-square test was used, with a *p*-value of less than 0.05 considered statistically significant. Minor missing values within completed questionnaires were handled using pairwise deletion during statistical analysis to preserve available data.

## Results

A total of 474 mothers participated in the final analysis. Of these, 44.7% were between the ages of 26 and 30. Around 35.4% of the mothers held a primary level of education, while 48.1% were employed. Additionally, 39.7% of the mothers reported their monthly income as partially sufficient. Regarding family size, 48.5% had two to three children, and 49.4% had children between the ages of one day and less than twelve months (Table 1).

**Table 1:** Socio-demographic characteristics of the study participants.

Characteristic	Frequency (*n* = 474)	Percentage (%)
Age of mothers		
18–25 years	172	36.3
26–30 years	212	44.7
31–34 years	52	11.0
35 years or older	38	8.0
Educational level		
Primary	168	35.4
Secondary	158	33.3
University	38	8.0
Postgraduate studies	110	23.2
Job title		
Student	172	36.3
Employee	228	48.1
Housewife	74	15.6
Monthly income		
Enough income	122	25.7
Partially sufficient income	188	39.7
Not enough income	164	34.6
Number of children		
One child	180	38.0
Two to three	230	48.5
More than three	64	13.5
Age of the children		
One day to less than 12 months	234	49.4
Twelve to less than 24 months	136	28.7
Two to less than five years	30	6.3
Five to less than 10 years	74	15.6

Descriptive statistics including frequency and percentage distributions were employed to summarize socio-demographic characteristics.

Table 2 presents the participants’ responses to the knowledge-based questions. It was found that 76.8% of mothers were aware that polio vaccination is essential for their children. Additionally, 73.0% acknowledged that polio vaccination reduces the risk of death or disease in children, while 66.7% understood its role in overall child health. Furthermore, 71.7% were aware that polio vaccinations are administered at specific ages.

**Table 2:** The study participants’ responses to the knowledge questions.

Knowledge questions	Frequency (*n* = 474)	Percentage (%)
1. Do you know that polio vaccination is necessary for your children?		
Yes	364	76.8%
No	110	23.2%
2. Did you know that polio vaccination reduces the risk of death or disease for a child?		
Yes	346	73.0%
No	128	27.0%
3. Did you know that polio vaccinations play a role in a child's health?		
Yes	316	66.7%
No	158	33.3%
4. Did you know that polio vaccinations have a certain age?		
Yes	340	71.7%
No	134	28.3%
5. Did you know that there are many types of polio vaccinations?		
Yes	322	67.9%
No	152	32.1%
6. Did you know that some vaccinations have side effects such as fever?		
Yes	292	61.6%
No	182	38.4%
7. Did you know that some vaccinations cause cramps and rashes?		
Yes	302	63.7%
No	172	36.3%
8. Did you know that low fever and diarrhea are contraindications to vaccination?		
Yes	296	62.4%
No	178	37.6%
9. Do you realize that even a healthy child needs to be vaccinated?		
Yes	300	63.3%
No	174	36.7%
10. Have you read about polio vaccinations before?		
Yes	320	67.5%
No	154	32.5%

Descriptive statistics, including frequency and percentage distributions were employed to summarize knowledge scores.

Moreover, 67.9% of the mothers were informed that there are different types of vaccines. A further 61.6% knew that some vaccines could cause side effects such as fever, and 63.7% were aware that certain vaccines could lead to cramps and rashes. Additionally, 62.4% understood that conditions like low fever and diarrhea are contraindications for vaccination. Lastly, 63.3% recognized the importance of vaccinating even healthy children, and 67.5% had previously read about vaccinations.

The analysis of participants’ attitudes and practices toward polio vaccination revealed the following: 75.5% of mothers expressed a positive view on the benefits of polio vaccination, 69.6% believed in its safety for children, 68.8% supported compulsory vaccination programs, and 67.5% encouraged their relatives and family members to have their children vaccinated. In terms of practices, 67.5% of the mothers reported that their children received mandatory polio vaccinations, 68.4% adhered to the vaccination schedules, 65.8% actively sought additional vaccines for their children, and 66.7% used pain relievers to alleviate swelling and discomfort post-vaccination (Table 3).

**Table 3:** Participants’ attitudes and practices towards polio vaccination.

Aspect	Frequency (*n* = 474)	Percentage (%)
Attitudes towards polio vaccination		
1. Do you think that polio vaccinations are beneficial?		
Yes	358	75.5%
No	116	24.5%
2. Do you feel that it is safe to vaccinate your child?		
Yes	330	69.6%
No	144	30.4%
3. Do you support the compulsory polio vaccination programs designed by the Ministry of Health?		
Yes	326	68.8%
No	148	31.2%
4. Do you advise your relatives and family to vaccinate their children?		
Yes	320	67.5%
No	154	32.5%
Practices related to polio vaccination		
1. Has your child received mandatory childhood polio vaccinations?		
Yes	320	67.5%
No	154	32.5%
2. Do you follow the mandatory polio vaccination programs included in the vaccination schedule?		
Yes	324	68.4%
No	150	31.6%
3. Are you looking for other vaccines available for your child?		
Yes	312	65.8%
No	162	34.2%
4. Do you use pain relievers to reduce swelling and pain after vaccinating your child?		
Yes	316	66.7%
No	158	33.3%

Descriptive statistics, including frequency and percentage distributions were employed to summarize attitudes and practices scores.

**Figure 1:**
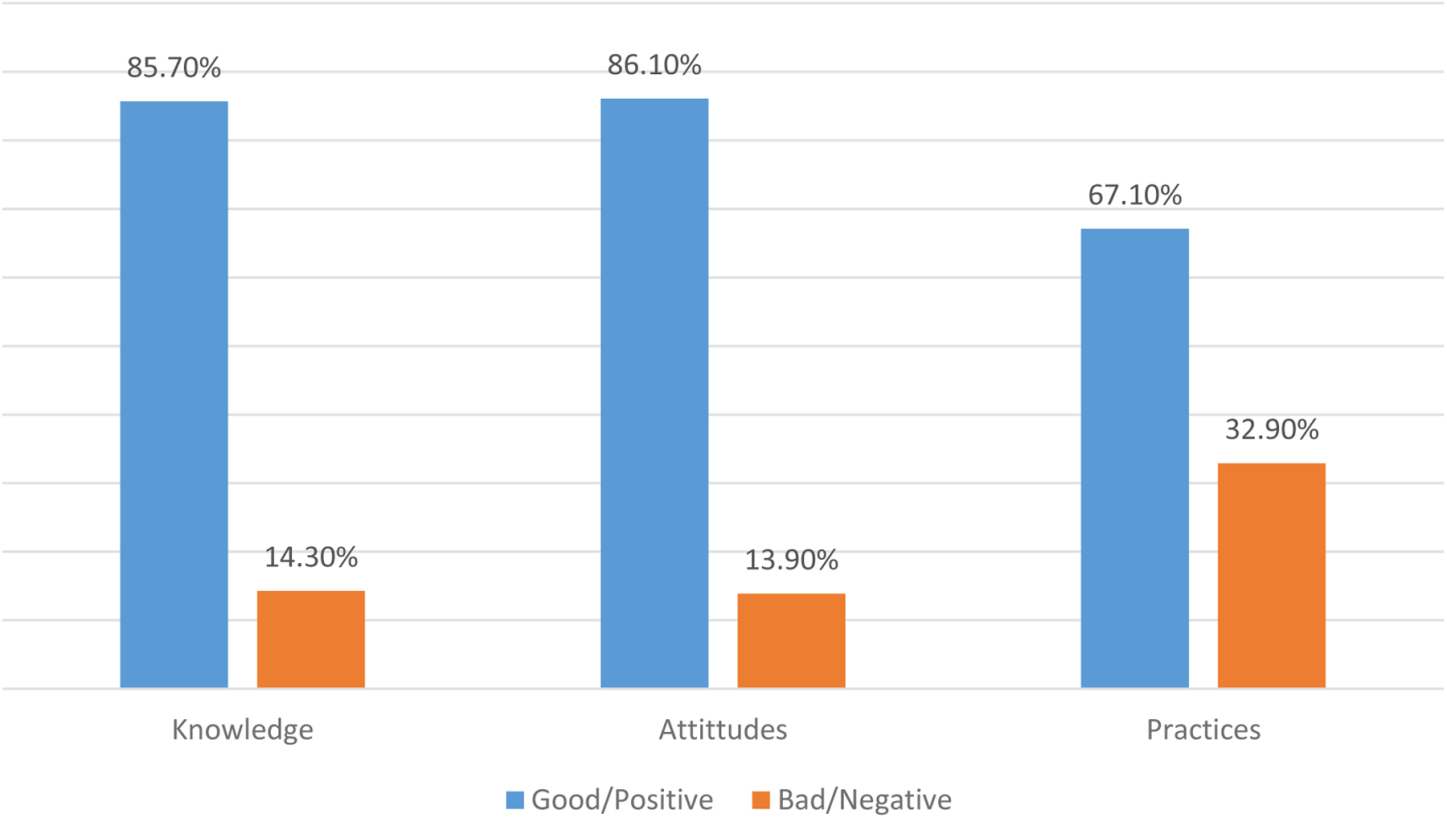
Total scores of knowledge, attitudes, and practices of mothers regarding polio vaccination.

The results indicated that 85.7% of the mothers demonstrated a strong understanding of polio vaccination. Additionally, 86.1% of the mothers held positive attitudes towards polio vaccination, and 67.1% exhibited effective practices related to it (Figure 1).

Table 4 demonstrates that the overall knowledge scores of the mothers were significantly associated with their age as well as the number and age of their children (*P*-values < 0.05). Specifically, younger mothers and those with younger children exhibited higher knowledge scores. Attitude scores were significantly related to both the mothers’ age and the number of their children (*P*-values < 0.05), with middle-aged mothers showing more positive attitudes. However, there was no significant association between any socio-demographic factors and the mothers’ practice scores (*P*-values > 0.05).

**Table 4:** Association between socio-demographic characteristics and total scores of knowledge, attitude, and practices.

Variables	Total knowledge			Total attitudes			Total practices		
	Poor	Good	*P*-value	Negative	Positive	*P*-value	Poor	Good	*P*-value
	*N* = 68 (%)	*N* = 406 (%)		*N* = 66 (%)	*N* = 408 (%)		*N* = 156 (%)	*N* = 318 (%)	
Age of mothers									
18–25 years	24 (35.3)	210 (51.7)	0.002*	12 (18.2)	160 (39.2)	0.004*	42 (26.9)	130 (40.9)	0.075
26–30 years	18 (26.5)	118 (29.1)		38 (57.6)	174 (42.6)		72 (46.2)	140 (44.0)	
31–34 years	4.0 (5.9)	26 (6.4)		16 (24.2)	36 (8.8)		24 (15.4)	28 (8.8)	
35 years or older	22 (32.4)	52 (12.8)		0.0 (0.0)	38 (9.3)		18 (11.5)	20 (6.3)	
Educational level									
Primary	16 (23.5)	152 (37.4)	0.235	18 (27.3)	150 (36.8)	0.489	50 (32.1)	118 (37.1)	0.351
Secondary	22 (32.4)	136 (33.5)		26 (39.4)	132 (32.4)		52 (33.3)	106 (33.3)	
University	4.0 (5.9)	34 (8.4)		8.0 (12.1)	30 (7.4)		10 (6.4)	28 (8.8)	
Postgraduate studies	26 (38.2)	84 (20.7)		14 (21.2)	96 (23.5)		44 (28.2)	66 (20.8)	
Job title									
Student	20 (29.4)	152 (37.4)	0.221	16 (24.2)	156 (38.2)	0.247	58 (37.2)	114 (35.8)	0.421
Employee	40 (58.8)	188 (46.3)		38 (57.6)	190 (46.6)		82 (52.6)	146 (45.9)	
Housewife	8.0 (11.8)	66 (16.3)		12 (18.2)	62 (15.2)		16 (10.3)	58 (18.2)	
Monthly income									
Enough income	18 (26.5)	104 (25.6)	0.356	10 (15.2)	112 (27.5)	0.541	34 (21.8)	88 (27.7)	0.224
Partially sufficient income	34 (50.0)	154 (37.9)		32 (48.5)	156 (38.2)		60 (38.5)	128 (40.3)	
Not enough income	16 (23.5)	148 (36.5)		24 (36.4)	140 (34.3)		62 (39.7)	102 (32.1)	
Number of children									
One child	10 (14.7)	170 (41.9)	0.001*	10 (15.2)	17 (4.2)	0.021*	54 (34.6)	126 (39.6)	0.614
Two to three	44 (64.7)	186 (45.8)		42 (63.6)	188 (46.1)		80 (51.3)	150 (47.2)	
More than three	14 (20.6)	50 (12.3)		14 (21.2)	50 (12.2)		22 (14.1)	42 (13.2)	
Age of the children									
One day to <12 months	24 (35.3)	210 (51.7)	0.004*	18 (27.3)	216 (52.9)	0.588	78 (50.0)	156 (49.1)	0.246
Twelve to <24 months	18 (26.5)	118 (29.1)		26 (39.4)	110 (27.0)		40 (25.6)	96 (30.2)	
Two to <five years	4.0 (5.9)	26 (6.4)		6.0 (9.1)	24 (5.9)		10 (6.4)	20 (6.3)	
Five to <10 years	22 (32.4)	52 (12.8)		16 (24.2)	58 (14.2)		28 (17.9)	46 (14.5)	

Descriptive statistics including frequency distributions were employed to summarize KAP scores. The Chi-square test was used to assess associations between categorical variables, with a * *p*-value of less than 0.05 considered statistically significant.

## Discussion

Understanding the factors that influence childhood immunization is pivotal for improving vaccination rates and safeguarding public health. Among these factors, mothers’ knowledge, attitudes, self-efficacy, and environmental conditions are critical variables that can be modified to enhance vaccination outcomes (17). This study aimed to assess the knowledge, attitudes, and practices (KAP) of mothers regarding polio vaccination at primary healthcare centers in the Gaza Strip during a period of conflict. The findings provide a nuanced view of the current state of polio vaccination among mothers in this challenging context and allow for comparisons with studies conducted in other countries.

The study found that a majority of the participating mothers demonstrated good knowledge and positive attitudes toward polio vaccination. This is promising, as it indicates a strong foundational understanding of the importance of immunization. The results are consistent with similar studies conducted in various countries. For instance, Adefolalu et al. found that 72.0% of participants in Nigeria had good knowledge about childhood immunization, with maternal age being a significant factor in knowledge levels (18). Similarly, Shati et al. reported that more than half of the mothers in Saudi Arabia had good knowledge about vaccination, which was notably associated with the number of children (19). These findings suggest that while knowledge is generally high, there are variations based on demographic factors.

In our study, younger mothers and those with younger children had higher knowledge scores. This finding aligns with research from other settings. For example, a study conducted in southeastern United States by Bradshaw et al. found that younger mothers were more likely to be informed about vaccination schedules, potentially due to higher engagement with digital health resources (20). Additionally, the study by Dhakal et al. in Nepal revealed that mothers with younger children had better knowledge of vaccination due to recent health education campaigns targeting new parents (21).

Regarding attitudes, the study observed that a majority of the mothers held positive views on polio vaccination. This finding is supported by similar research conducted globally. Verulava et al. in Georgia and Shati et al. in Saudi Arabia also found that mothers had a positive attitude toward immunization, viewing it as a crucial tool for disease prevention (19, 22). This is encouraging as positive attitudes are often correlated with higher vaccination uptake. In contrast, a study in the United States by Bardenheier et al. found that despite positive attitudes, vaccine hesitancy due to misinformation and safety concerns was prevalent, impacting overall vaccination rates (23).

Our study identified that middle-aged mothers exhibited particularly strong positive attitudes. This is in line with the findings of Mohammed and Al-Zahrani, who reported that middle-aged mothers in Saudi Arabia had more positive attitudes towards vaccination compared to younger mothers (24). This could be attributed to their accumulated experience and exposure to various public health initiatives over time.

In terms of practices, the study revealed that about two-thirds of the mothers exhibited good practices related to polio vaccination, such as adhering to mandatory vaccination programs and seeking additional vaccines. This result is comparable to findings from Saudi Arabia, where Alyami et al. reported that 62% of participants adhered to recommended immunization schedules (25). However, a study in Brazil by Lima et al. found a disparity between high levels of knowledge and low vaccination uptake due to concerns about vaccine safety and trust issues (26). This discrepancy underscores the complexity of translating knowledge and positive attitudes into effective vaccination practices.

The current study also noted a lack of significant correlation between socio-demographic factors and vaccination practices. This finding contrasts with studies such as those by Alyami et al. and Mohammed and Al-Zahrani, which found that factors such as higher education, older age, and greater knowledge were positively associated with better vaccination practices (24, 25). In our context, the ongoing conflict and limited healthcare resources may overshadow these socio-demographic influences, highlighting the need for context-specific interventions.

Comparing the results of this study with those from other countries reveals several key insights. While knowledge and attitudes towards polio vaccination are generally positive across different settings, the translation of these factors into effective vaccination practices varies. In high-resource settings, such as the United States and Saudi Arabia, positive attitudes and knowledge often lead to higher vaccination rates. In contrast, in conflict-affected or low-resource settings like Gaza and parts of Brazil, barriers such as safety concerns, logistical challenges, and mistrust can impede the translation of knowledge and positive attitudes into practice.

The findings from Gaza underscore the need for targeted interventions that address both informational needs and practical barriers to vaccination. In conflict settings, where access to healthcare and resources may be limited, it is crucial to develop strategies that not only enhance knowledge and attitudes but also address the specific challenges faced by mothers in these environments. This could involve strengthening healthcare infrastructure, providing reliable information about vaccine safety, and improving accessibility to vaccination services.

This study underscores the importance of primary healthcare centers as key access points for immunization, even during conflict. However, to reach mothers not attending these centers, strategies such as community outreach, mobile vaccination units, and targeted health education are essential. Integrating immunization with broader maternal health services and strengthening health system resilience through workforce training and secure vaccine supply chains are critical for sustaining vaccination efforts in crisis settings. Due to the widespread disruption of health centers caused by the recent conflict in the Gaza Strip, maintaining access to vaccination services necessitates practical strategies. These include enabling support from international medical aid organizations, creating humanitarian corridors to ensure the safe delivery of vaccines, utilizing community spaces as temporary vaccination sites, and employing digital technologies to track immunization coverage, manage logistics, and facilitate communication with impacted communities. Finally, bridging the gap between maternal knowledge and vaccination practices in Gaza requires a comprehensive approach that improves access, strengthens health education, empowers healthcare workers, integrates services, engages community networks, and ensures strong policy support and resource coordination.

## Strength and limitations

This study has several strengths and limitations that should be considered when interpreting the findings.

### Strengths

One of the primary strengths of this study is its novel contribution to understanding the knowledge, attitudes, and practices (KAP) of mothers regarding polio vaccination in the Gaza Strip during a period of conflict. As the first study to focus on this topic in such a challenging context, it provides valuable insights into how a war environment impacts maternal perceptions and behaviors related to immunization. Additionally, the study's use of a representative sample enhances the reliability of the findings, making them more applicable to the broader population of mothers in the Gaza Strip.

### Limitations

The study was conducted among women who visited vaccination sites, so they likely already held some positive attitudes toward vaccinations. The study's cross-sectional design is a notable limitation, as it captures data at a single point in time, which can affect the generalizability of the results. This design does not allow for the examination of changes over time or the establishment of causal relationships between variables. Furthermore, the study focused exclusively on mothers, potentially excluding the perspectives of other key caregivers, such as fathers or extended family members, who may also influence vaccination decisions. This narrow focus may limit the comprehensiveness of the understanding of factors affecting childhood immunization. Additionally, given that data were collected exclusively from mothers attending immunization services at selected primary healthcare centers, the results may not be generalizable to mothers who do not utilize these services. This may introduce selection bias, as those who attend vaccination sites might differ in their KAP from those who do not, particularly in the context of ongoing conflict and access challenges.

While this study provides important insights into maternal KAP regarding polio vaccination in a conflict zone, the limitations related to its design and participant scope should be acknowledged and addressed in future research to provide a more holistic view of vaccination practices and perceptions.

Some mothers, who appeared to hold negative attitudes yet still attended vaccination sites, could have benefited from targeted health communication to address vaccine reluctance caused by misinformation, fear, or cultural resistance. This highlights the importance of building trust and providing continuous support from healthcare providers to alleviate negative attitudes, ultimately boosting mothers’ confidence in immunization and ensuring better protection for their children.

## Conclusion

The findings indicate that the majority of mothers in the Gaza Strip possess a strong knowledge base and positive attitudes towards polio vaccination. However, despite this high level of awareness and favorable attitudes, approximately two-thirds of the mothers demonstrated only good practices, suggesting a gap between knowledge and application. Younger mothers and those with younger children generally exhibited better knowledge, while middle-aged mothers were more likely to hold positive attitudes towards vaccination. Notably, socio-demographic factors did not show a significant relationship with the practice levels observed. The observed discrepancy between knowledge and actual vaccination practices highlights an area of concern that warrants attention. To address these issues, it is crucial to conduct further research in various regions and among diverse populations to better understand and generalize the findings. Additionally, exploring the influence of cultural and socioeconomic factors on vaccination knowledge, attitudes, and practices could provide valuable insights for developing targeted interventions. Tailoring strategies to address specific contextual factors will be essential for improving vaccination practices and ensuring more effective public health outcomes.

## Data Availability

The raw data supporting the conclusions of this article will be made available by the authors, without undue reservation.
